# 3-Hydroxy-7,8,9,10-tetrahydro-6H-benzo[c]chromen-6-one and 3-hydroxy-6H-benzo[c]chromen-6-one act as on-off selective fluorescent sensors for Iron (III) under in vitro and ex vivo conditions

**DOI:** 10.3906/kim-2011-58

**Published:** 2021-06-30

**Authors:** Karar SHUKUR, Amirhossein FALLAH, Kerem TERALI, Rasime KALKAN, Mustafa GAZI, Hayrettin Ozan GÜLCAN

**Affiliations:** 1 Department of Pharmaceutical Chemistry, Faculty of Pharmacy, Eastern Mediterranean University, Gazimağusa Turkish Republic of Northern Cyprus; 2 Department of Chemistry, Faculty of Art and Science, Eastern Mediterranean University, Gazimağusa Turkish Republic of Northern Cyprus; 3 Department of Medical Biochemistry, Faculty of Medicine, Near East University, Lefkoşa Turkish Republic of Northern Cyprus; 4 Department of Medical Genetics, Faculty of Medicine, Near East University, Lefkoşa Turkish Republic of Northern Cyprus

**Keywords:** Urolithin, fluorescence probe, on-off sensor, Iron (III)

## Abstract

Regarding the abundant use of metals for different purposes, it becomes more critical from various scientific and technological perspectives to discover novel agents as selective probes for the detection of specific metals. In our previous studies, we have shown that aqueous solutions of natural urolithins (i.e., hydroxyl-substituted benzo[c]chromen-6-one derivatives) are selective Iron (III) sensors in fluorescence assays. In this study, we have extrapolated these findings to another coumarine compound (i.e., 3-Hydroxy-7,8,9,10-tetrahydro-6H-benzo[c]chromen-6-one) and compared the selective metal binding properties with Urolithin B (i.e., 3-Hydroxy-6H-benzo[c]chromen-6-one). Following the synthesis and structure identification studies, the fluorometric studies pointed out that the lactam group in the structure still persists to be the important scaffold for maintaining selective on-off sensor capacity that renders the compound a selective Iron (III) binding probe. Moreover, for the first time, fluorescence cellular imaging studies concomitant to cytotoxicity assays with the title compounds were also performed and the results displayed the cell-penetrative, safe, and fluorescent detectable characteristics of the compounds in neuroblastoma and glioblastoma cells through servings as intracellular Iron (III) on-off sensors.

## 1. Introduction

The design or discovery of small molecules capable of detecting metals continues to attract curiosity in relation to the abundant employment of various metals in science and technology and the resulting accumulation and pollution in environment [1–2]. In fact, extensive environmental exposure to these metals by living things, including humankind, can lead to serious damages to health. At one side, many metals are utilized in regular physiological activities such as the ones employed in some enzyme-catalyzed reactions [3–4]. On the other hand, severe disease states, acute or chronic toxicities can be formed through uncontrolled and overdose exposure to metals [5]. Although many mechanisms have been proposed, particularly Fenton-like reactions triggered by metals constitute the basic toxic effects resulting in the formation of oxidized cellular macromolecules, such as lipids and proteins [6]. 

Among metals, iron has special properties with respect to its physiological functions and its environmental characteristics [7]. Indeed, iron has important activities in humans such as the ones in oxygen transportation, mitochondrial respiration, neurotransmitter synthesis, myelin synthesis, DNA synthesis, and cellular metabolic activities [7–8]. However, it can also act as a catalyst leading to the generation of highly toxic free radicals. Therefore, depending on its concentration, iron has double-edged sword effects on human homeostasis. For instance, a growing body of evidence suggests a causal role of this metal in neurodegenerative disorders such as Alzheimer’s disease and Parkinson disease; therefore, monitoring or detecting brain iron accumulation have been suggested to be useful both in the early diagnosis of neurodegenerative diseases and in the prognosis of affected patients [9–10]. 

There have been many studies conducted on the discovery of small molecules or polymers acting as selective sensors to detect iron at different oxidation states. In our recent studies, we have demonstrated that urolithins (i.e., hydroxylated benzo[c]chromen -6-one derivatives) can act as fluorescent chemosensors for the selective detection of Iron (III) in aqueous solutions [11–12]. Urolithins are the bioavailable metabolites of an ellagitannin-rich diet including pomegranate, nuts, and berries. Gastrointestinal microflora directed metabolism of ellagitannins generate urolithin derivatives, and they act as biomarkers of ellagitannin exposure in systemic circulation [13–14]. 

According to our previous findings, the lactone group in urolithin structure is the key group for the selective interaction with Iron (III), since the number of phenolic hydroxyls and their conversion to methyl ethers have been shown to be independent on this feature [12]. Based on this information, in the present study, 3-hydroxy-7,8,9,10-tetrahydro-6H-benzo[c]chromen-6-one (i.e., THU-OH), a partially saturated analogue of Urolithin B (i.e., 3-hydroxy-6H-benzo[c]chromen-6-one, URO-B), was synthesized, and its characteristics to interact with metals were aimed to be investigated concomitant to the comparison of the results obtained for Urolithin B under the same experimental conditions. Finally, the cellular fluorescence imaging, and cytotoxicity assays were also designed to evaluate membrane penetration characteristics of these molecules, their safety, and on-off fluorescence sensor characteristics in cell-based experiments.

## 2. Experimental analysis

### 2.1. Materials and equipment

All reagents and solvents used for synthesis, fluorometric measurements, and cell-based assays were of reagent grade. Ethyl 2-cyclohexancarboxylate, resorcinol, 2-iodobenzoic acid, zirconium chloride, acetonitrile, dichloromethane, mono-, di-, and tri-valent metal salts (KCl, NaCl, AgCl, CuI, FeCl3, PbSO4, BaSO4, CuSO4, ZnSO4.7H2O, CaCl2, Co(NO3)2, FeSO4.7H2O, BBr3, Al2(SO4)3, MgSO4.7H2O, Ni(NO3)2, HgSO4) were purchased from Sigma-Aldrich, (Sigma-Aldrich Corp., St. Louis, MO, USA). Stock solutions of metal ions were prepared with ultrapure water. 

Fluorometric measurements were performed employing a 96-well microplate fluorescence spectroscopy reader (Varioskan Flash, Thermo Fisher Scientific Inc., Waltham, MA, USA). The cytotoxicity assay based measurements were completed using a tunable UV microplate reader (VERSA max, Molecular Devices LLC, Sunnyvale, CA, USA). The cell imaging studies were achieved using a fluorescence microscope (AXIO Imager.M2, Zeiss Microscopy GmbH, Jena, Germany). 

To monitor the reactions, thin layer chromatography (TLC) was performed on Merck aluminum-packed silica gel plates employing Ethyl acetate – n-hexane (2:1). The melting points of the compounds were determined using an Electrothermal IA 9200 Model meting point apparatus, and the results found were uncorrected. A Shimadzu FT-IR Prestige 21 model spectrophotometer was used for infrared studies. NMR studies were conducted on a Bruker-400 NMR spectrometer. Tetramethylsilane was used as internal standard, and the title compounds were dissolved in DMSO-d_6_. The chemical shifts were reported in parts per million (ppm, δ). A ThermoFisher Flash Smart CHNS elemental analyzer was used to measure elemental analysis.

### 2.2. Synthesis of 3-hydroxy-7,8,9,10-tetrahydro-6H-benzo[c]chromen-6-one (THU-OH)

10 mmol of resorcinol and and 11 mmol of ethyl 2-cyclohexancarboxylate was heated at 85 °C under neat conditions for 30 min in the presence of 7.5 mmol of ZrCl_4_. At the end of the period, the precipitate formed was added into 20 mL of ice-cold water. Following the filtration, the product was obtained as light-yellow powder. Melting point: 209 ºC (uncorrected data). ^1^H NMR (DMSO d6,400 MHz): δ = 10.31 (s, 1H), 7.46 (d, 1H, 7,2Hz), 6.74 (d,1H, 6,8Hz), 6.65 (s, 1H), 2.68 (t, 2H, 4,0Hz), 2,34 (t, 2H, 4.4Hz), 1,7 (m, 4H). ^13^C NMR (DMSOd6, 125 MHz) δ (ppm) major rotamer: 161.0 ppm for lactone carbonyl carbon. IR: 1678,7 cm^−1^ for lactone carbonyl. Yield obtained: 81%. Anal. calc. for C13H12O3: C 72.21, H 5.59; found C 72.44, H 5.67.

### 2.3. Synthesis of 3-hydroxy-6H-benzo[c]chromen-6-one (URO-B)

Resorcinol (45mmol), 2-iodobenzoic acid (15mmol), and 55mmol of NaOH were dissolved in 30 mL of distilled water. The solution was refluxed for 45min. At the end of the period, the brown solution formed was added 6g of CuSO_4_. Following an additional 10 min reflux of the solution, the mixture was cooled. The product precipitated was filtered off and washed with cold 100mL of 0.1N HCl solution and it was obtained as yellow powder. Melting point: 228 ºC (uncorrected data). ^1^H NMR (DMSO d6,400 MHz): δ = 10.33 (s, 1H), 8,17 (m, 3H), 7.87 (t,1H, 5,2Hz), 7.52 (s, 1H), 6.82-6.74 (m, 2H). ^13^C NMR (DMSOd6, 125 MHz) δ (ppm) major rotamer: 187.5 ppm for lactone carbonyl carbon. IR: 1691,4 cm^−1^ for lactone carbonyl. Yield obtained: 79%. Anal. calc. for C13H8O3: C 73.58, H 3.80; found C 73.70, H 3.91.

### 2.4. Fluorometric studies and titrations

The excitation and emission wavelengths of THU-OH was identified in acetonitrile water (ACN:H2O, 1:1, v/v) solution at a 500 nm/min scan rate. For the titration studies, the selected concentrations of metal ions in ACN:H_2_O (1:1, v/v) were added to the solutions of THU-OH or URO-OH. Mainly, 0.5 mM stock solutions of the title molecules were prepared in 100 mL of 1:1 ACN-H_2_O solution. In order to determine Iron (III) detection, 1 mM Iron (III) stock solution was prepared in100 mL ACN/H2O (1:1 v/v). Finally, known concentrations of Iron (III) aqueous solution was then added into the probe solution and thoroughly mixed for 10 min, followed by the fluorescence measurement. Metal ions were prepared from various salts of K+, Na+, Cu+, Ag+, Ba+, Ca2+, Zn2+, Co2+, Ni2+, Hg2+, Cu2+, Fe2+, Fe3+, B3+, and Al3+ under the same conditions to screen selectivity. 

### 2.5. Cell proliferation and cytotoxicity assays

The cytotoxic effects of the URO-B and THU-OH probes (both dissolved in 100% acetonitrile at a stock concentration of 20 mM) on human neuroblastoma (SK-N-AS, ATCC, Manassas, VA, USA) and glioblastoma (DBTRG-05MG, ATCC, Manassas, VA, USA) cells were determined by using Cell Counting Kit (CCK)-8 (Dojindo EU GmbH, Munich, Germany), which was based on the reduction of the WST-8 tetrazolium salt by dehydrogenases inside viable cells to yield a water-soluble formazan dye (orange color). Here, 100 μL of cell suspension in complete growth medium (RPMI-1640 supplemented with 10% fetal bovine serum, 1% L-glutamine, and 1% penicillin–streptomycin) was dispensed in a 96-well plate at 5,000 cells/well, and the plate was pre-incubated for 24 h in a humidified incubator (i.e. at 37 °C with 5% CO2 and 95% air). 10 μL of URO-B or THU-OH at a given working concentration was added to the plate to achieve six different test concentrations of 6.25 mM, 12.5 mM, 25 mM, 50 mM, 100 mM, and 200 mM. The plate was incubated for 24 h in the incubator, and 10 μL of CCK-8 solution was added to each well of the plate after the incubation perion was over. The plate was further incubated for 4 h in the incubator, and the resulting absorbance at 450 nm was measured by using a tunable microplate reader (VERSA max, Molecular Devices LLC, Sunnyvale, CA, USA). The inhibition of cell proliferation was quantitated according to the instructions of Dojindo EU GmbH.

### 2.6. Fluorescence cell imaging

Human SK-N-AS (neuroblastoma) and DBTRG-05MG (glioblastoma) cells were separately seeded in 18 mm × 50 mm slide-flasks at 50,000 cells/mL. Following overnight incubation in a humidified incubator (i.e. at 37 °C with 5% CO2 and 95% air), SK-N-AS cells were treated with 50 μM URO-B (diluted with RPMI-1640) and DBTRG-05MG cells were treated with 50 μM THU-OH (diluted with RPMI-1640) for 1 h in the incubator. The final concentration of the co-solvent (acetonitrile) in the slide-flasks was 0.25%. The cells were washed twice with RPMI-1640 to remove the excess probe and subsequently treated with 50 mM Fe(NO_3_)_3_·9H_2_O (dissolved in RPMI-1640) for 2 h in the incubator. The upper flask structure was detached from the slide, and the free slide was briefly dried in the incubator. The cells were imaged under a fluorescence microscope (AXIO Imager.M2, Zeiss Microscopy GmbH, Jena, Germany) equipped with a 4′,6-diamidino-2-phenylindole (DAPI) filter set that had a UV excitation wavelength of 330–380 nm and an emission wavelength of 420 nm.

## 3. Results and discussion

### 3.1. Synthesis and characterization

**Figure 1:**
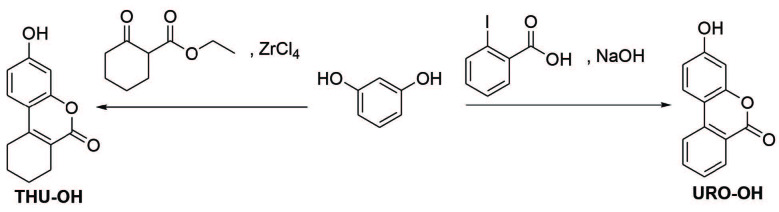
The synthesis of the title molecules.

Previously described methodologies were followed for the synthesis of the title molecules and the synthesis scheme of the title compounds is shown in Figure 1 [15–16]. Accordingly, resorcinol was used as the starting material for the synthesis of both THU-OH and URO-B. THU-OH was synthesized employing the Pechmann reaction conditions in good yield, as previously described [15]. On the other hand, URO-B was synthesized according to the classical synthetic methodology on urolithin derivatives [15–16]. The structures of the compounds were identified employing spectroscopic techniques and elemental analysis. Accordingly, the spectral data obtained is in parallel with the literature data [15–16]. 

### 3.2. Fluorescence spectral properties of the title compounds

**Figure 2:**
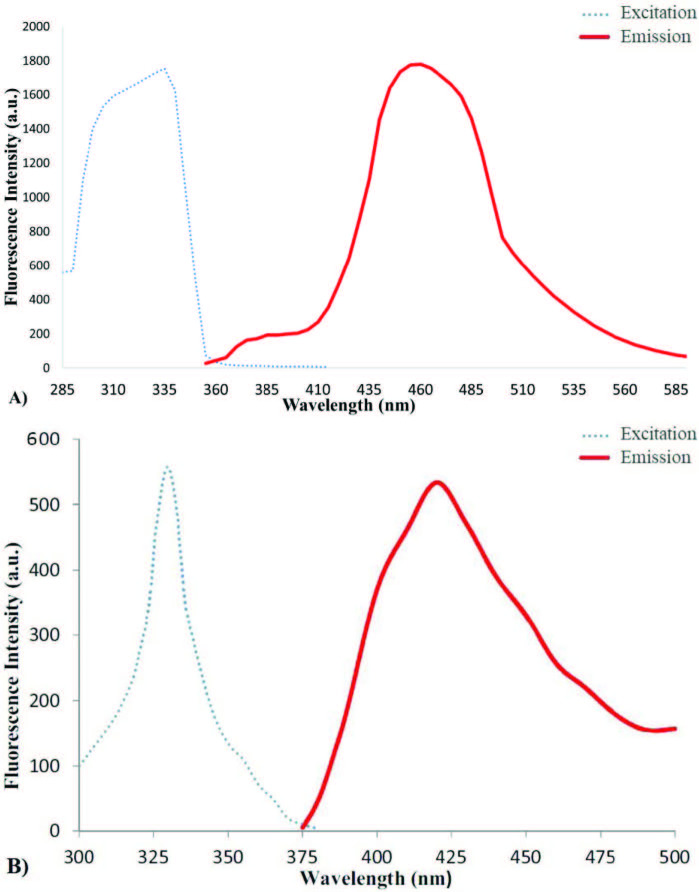
Excitation and emission spectra of THU-OH (A) and URO-B (B) in ACN/H2O (1:1 v/v).

The excitation and emission spectra of the title molecules in ACN/H_2_O (1:1 v/v) are shown in Figure 2. Accordingly, the excitation wavelengths respectively for THU-OH, and URO-B were determined as 335 and 330nm, while the emission wavelengths were found to be 460 and 420nm. Both spectra were observed to possess large Stokes shifts (i.e., 125 nm for THU-OH and 90nm for URO-B). 

### 3.3. Spectral responses of the title compounds against Iron (III)

**Figure 3:**
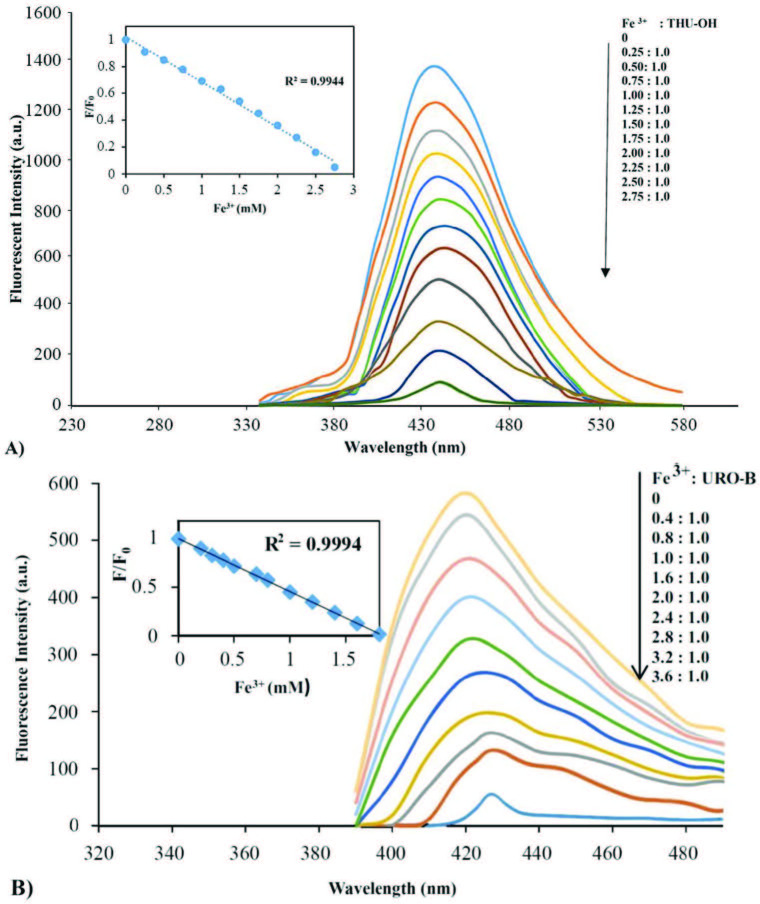
Fluorescence spectra of THU-OH (A) and URO-B (B) in the presence of various concentrations of Iron (III) in ACN/H2O (1:1 v/v).

The fluorescence changes of the title molecules with respect to the different concentrations of iron (III) were examined in ACN/H2O solution (1:1, v/v). The results are shown in Figure 3. Accordingly, it was observed that through the addition of various concentrations of iron (III), the intensity of fluorescence peak at 420nm for URO-B, and 460nm for THU-OH decreased in a proportional way. At 2.7mM of iron (III) concentration, the fluorescence was found almost totally quenched for THU-OH. On the other hand, 0.5mM concentration of Iron (III) made a total quenching of the fluorescence of URO-B. The linearity in terms of fluorescence quenching for both probes depending on increasing iron (III) concentrations is also displayed in the inset of Figure 3.

### 3.4. Selectivity of fluorescence response of the probes towards Iron (III)

**Figure 4:**
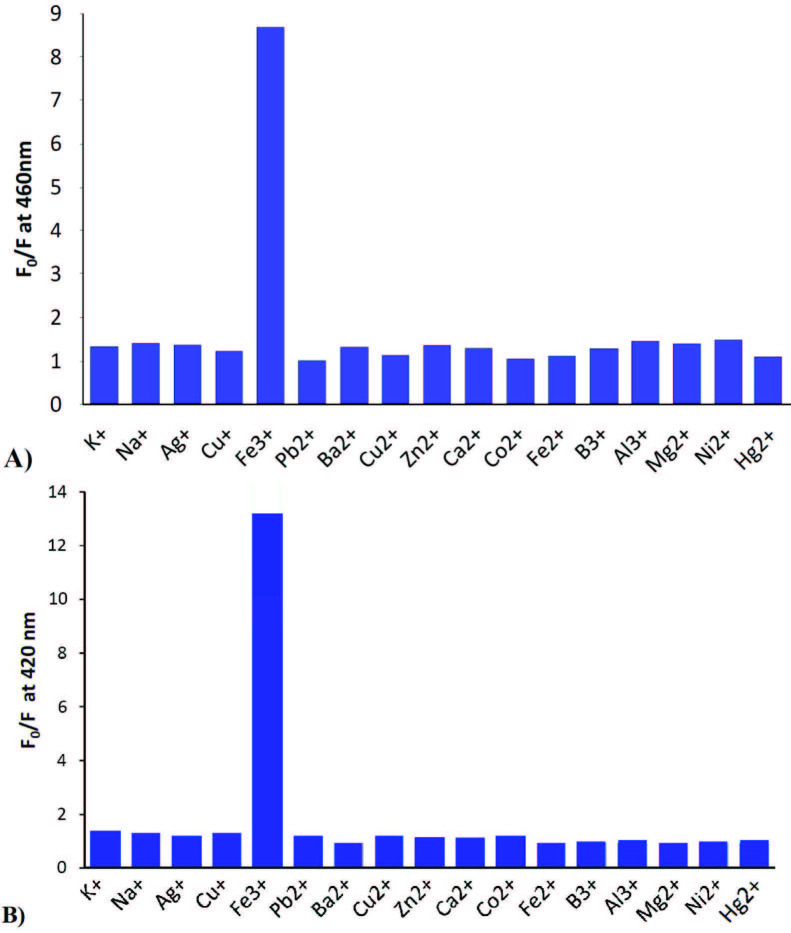
Selectivity of THU-OH (A) and URO-B (B) probes in the presence of different metal ion solutions in ACN/H2O (1:1 v/v).

Various metals with different charges (i.e., mono-, di-, and tri-valent metal ions) were employed to screen the selectivity of the probes prepared. For this group of studies, the solutions of the title molecules and metal cations were prepared at a 1:1 ratio in 1.5 mL of ACN/H_2_O (1:1, v/v). The results obtained are shown in Figure 4. As the results pointed out, the probes prepared were selective to iron, since no similar quenching effect was observed in the presence of metal ions employed. 

### 3.5. Interference study of the title compounds

**Figure 5:**
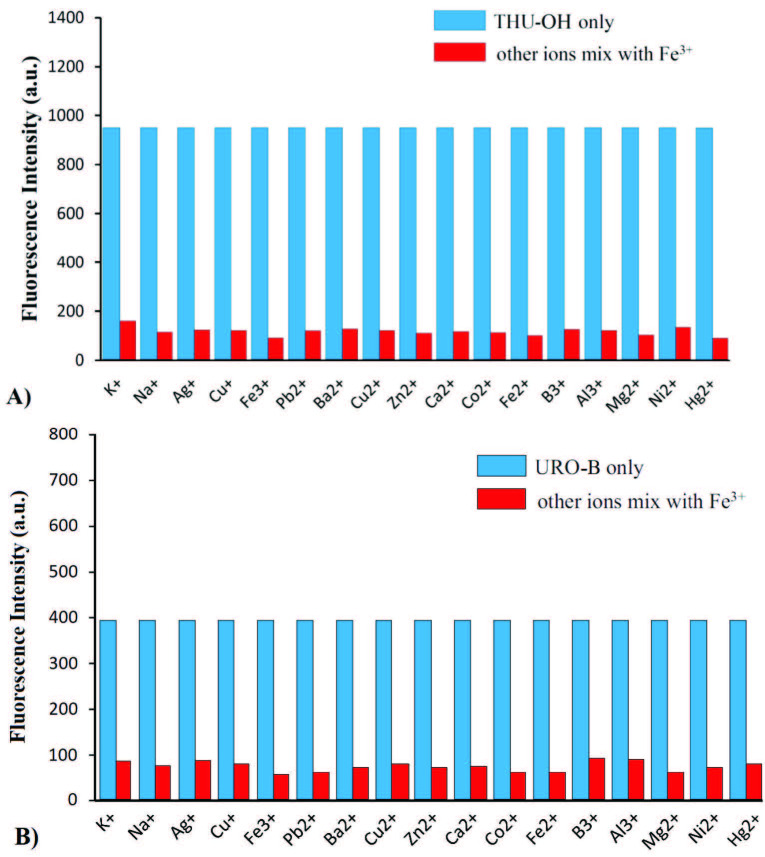
Selectivity of THU-OH (A) and URO-B (B) probes in the presence of different metal ion solutions in ACN/H2O (1:1 v/v).

In order to see the effect of interference, the quenching of title molecules (i.e., 0.1mM concentrations of both probes) with Iron (III) was examined in the presence of both iron (III) and other metal ions (i.e., 5 equivalents of K+, Na+, Ba2+, Ca2+, Zn2+, Co2+, Ni2+, Hg2+, Ag+, Cu2+, Cu+, Fe2+, Al3+, and B3+ with 3 equiv. of Fe3+). The results are displayed in Figure 5. As clearly demonstrated, no interference was observed in terms of quenching effect of iron (III) in the presence of other metal ions.

### 3.6. The stoichiometry of interaction with Iron (III)

In our previous studies on urolithin analogues, we have observed the importance of the lactone group in terms of interaction with iron (III). Considering the fact that both THU-OH and URO-B also possess the lactone moiety, the selective iron (III) quenching observed with the title molecules might be related to the lactone group as well. In other words, the de-aromatization of the ring in which the carbonyl carbon is directly attached, there has been observed limited or no effect on selective iron (III) interaction and consequent fluorescence quenching. However, it is noteworthy to state that the de-aromatization of the carbonyl carbon attached ring yielded out higher intensity in fluorometric measurements in comparison to the urolithin analogue (i.e., URO-B). 

**Figure 6:**
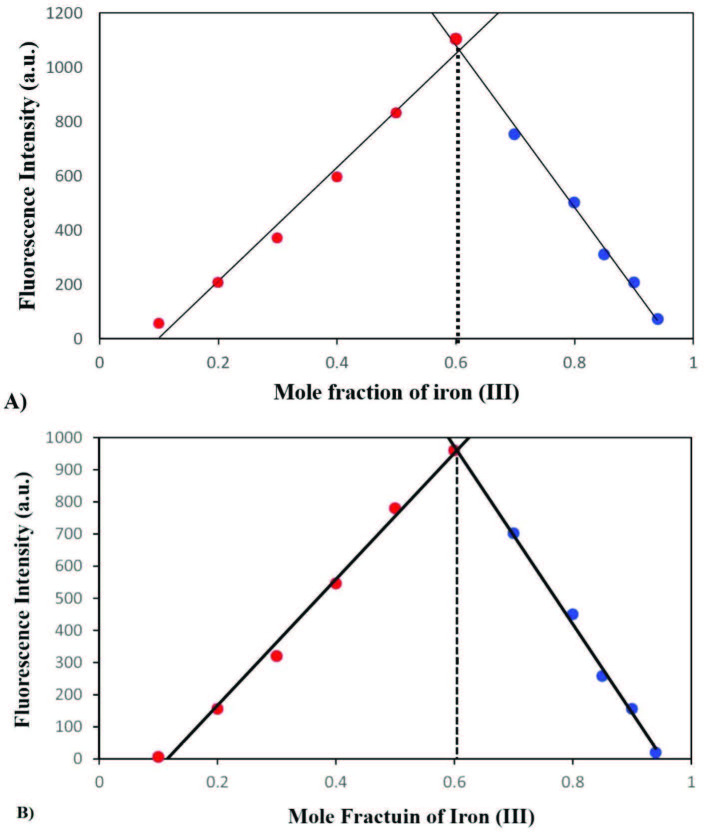
The Job’s plot for the coordination of THU-OH (A) and URO-B (B) with Iron (III) in ACN/H2O (1:1 v/v).

The Job’s plots were also employed to delineate the precise stoichiometry of the interaction between Iron (III) and the probes employed. As shown in Figure 6, the stoichiometry studies resulted in different ratios. For THU-OH, the stoichiometry was found to be 3:2 (THU-OH:Iron (III)). These results pointed out that the interaction of THU-OH is the same as the urolithin compounds (i.e., Urolithin A and B) as we have found in our previous studies [11-12]. 

### 3.7. Cytotoxicity and fluorescence cell imaging

**Figure 7:**
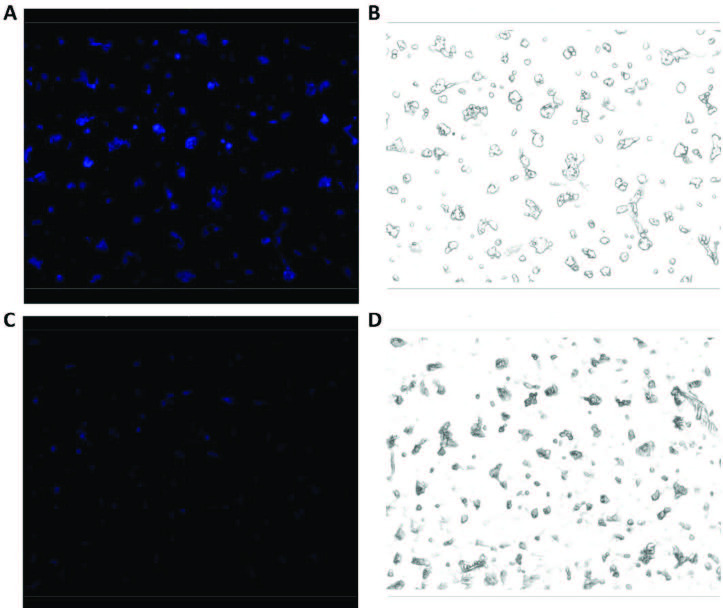
Fluorescence-quenching effect of Iron (III) on neuroblastoma cells incubated with URO-B. (A) Fluorescence microscopy image of SK-N-AS cells incubated with 50 μM URO-B. (B) Light microscopy image of the same region shown in A. (C) Fluorescence microscopy image of SK-N-AS cells after 50 mM Fe(NO3)3 9H2O treatment. (D) Light microscopy image of the same region shown in C. All images were collected with a 10  objective lens.

**Figure 8:**
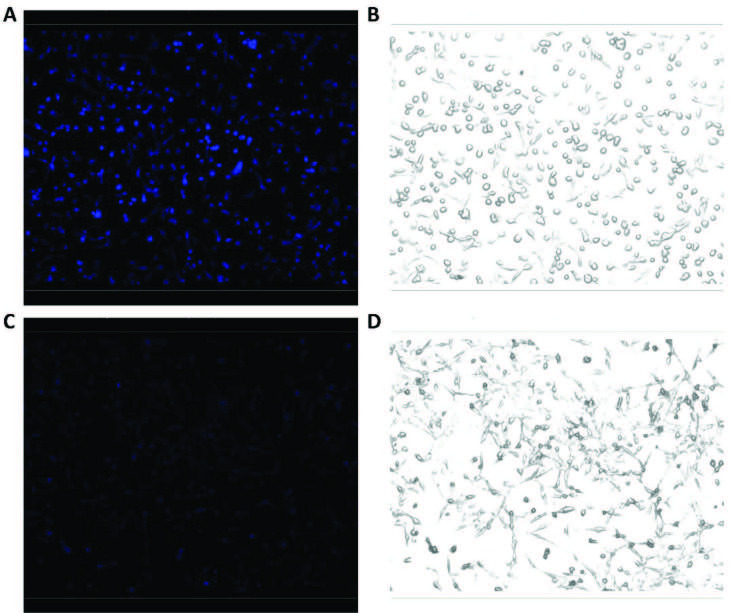
Fluorescence-quenching effect of Iron (III) on glioblastoma cells incubated with THU-OH. (A) Fluorescence microscopy image of DBTRG-05MG cells incubated with 50 μM THU-OH. (B) Light microscopy image of the same region shown in A. (C) Fluorescence microscopy image of DBTRG-05MG cells after 50 mM Fe(NO3)3 9H2O treatment. (D) Light microscopy image of the same region shown in C. All images were collected with a 10  objective lens.

The results of the cytotoxicity assays revealed that at 50 μM URO-B, 78% of human SK-N-AS (neuroblastoma) cells were viable. Similarly, 90% of human DBTRG-05MG (glioblastoma) cells survived in the presence of 50 μM THU-OH. Fluorescence microscopy experiments at the aforementioned probe concentrations demonstrated that both URO-B and THU-OH were able to readily penetrate the cells, due mainly to their lipophilic nature. SK-N-AS cells incubated with URO-B initially displayed a strong fluorescent signal, but the fluorescent signal quickly became faint and tenuous in the presence of iron (III) ions (Figure 7). The Iron (III) mediated “turn-off” behaviour was also observed for DBTRG-05MG cells incubated with THU-OH (Figure 8).

## 4. Conclusion

The results of the current study absolutely pointed out that 3-Hydroxy-7,8,9,10-tetrahydro-6H-benzo[c]chromen-6-one (THU-OH), a coumarine derivative and also a saturated Urolithin B analogue, serves as an Iron (III) selective fluorescent on-off sensor in an aqueous environment. This property of THU-OH was found to be independent from the presence of alternative metals in the surroundings. The stoichiometry in terms of its interaction with Iron (III) displayed its parallel features with the previously studies urolithin derivatives. From this perspective, it was clearly shown that the alpha-beta unsaturated lactone system present in urolithins plays a key role both in the generation of fluorescence properties and in the selectivity towards Iron (III). Similar studies with alternative synthetic analogues bearing coumarine moiety with an ester functional inside the ring pointed out the importance of the lactone group for the specific interaction with Iron (III) [17]. Although majority of the study in the relevant field focuses more on the development of OFF-ON type probes for selective Iron (III) detection, this type of approach through on-off strategy generates alternative probes [18–19]. 

Although there have been limited number of studies indicating cell viability supressing activity of some urolithin species particularly on human prostate cancer cells, within the context of the applicability to living-cell imaging in our studies, URO-B and THU-OH appear not to display significant cytotoxicity in the low-to-mid micromolar range, suggesting that they are biocompatible [20]. Furthermore, they have a high membrane permeation rate and maintain their Iron (III) binding activity in the intracellular environment, the latter of which is analogous to what is observed in in vitro experiments. Overall, our findings indicate that the urolithin-derived fluorescent probes can be suitable “turn-off” chemosensors for the fast and selective monitoring/detection of Iron (III) in biological systems. 

## References

[ref1] (2018). Chemically diverse small molecule fluorescent chemosensors for copper ion. Coordination Chemistry Reviews.

[ref2] (2018). Small-molecule fluorescent probes and their design. RSC Advances.

[ref3] (2013). copper in retinal physiology and disease. Survey of Ophthalmology.

[ref4] (2008). Metal ions in biological catalysis from enzyme databases to general principles. JBIC Journal of Biological Inorganic Chemistry.

[ref5] (2014). Toxicity, mechanism and health effects of some heavy metals: Interdisciplinary toxicology.

[ref6] (2011). Advances in metal-induced oxidative stress and human disease:. Toxicology.

[ref7] (2003). McKie A.T. Physiology and molecular biology of dietary iron absorption: Annual review of nutrition.

[ref8] (2005). Linking physiological functions of iron: Nature chemical biology.

[ref9] (1997). Iron accumulation in Alzheimer disease is a source of redox-generated free radicals:. Proceedings of the National Academy of Sciences.

[ref10] (2007). Individual dopaminergic neurons show raised iron levels in Parkinson disease. Neurology.

[ref11] (2018). B as a simple, selective, fluorescent probe for sensing Iron (III) in semi-aqueous solution. Journal of fluorescence.

[ref12] (2020). A and B Derivatives as ON-OFF Selective Fluorescent Sensors for Iron (III):. Journal of Fluorescence.

[ref13] (2018). synthesis and characterization of novel urolithin derivatives as cholinesterase inhibitor agents: Letters in Drug Design. Discovery.

[ref14] (2020). Molecular Docking, and Biological Activities of Some Natural and Synthetic Urolithin Analogs: Chemistry & Biodiversity.

[ref15] (2014). Design synthesis and biological evaluation of novel 6H-benzo [c] chromen-6-one.

[ref16] (2017). Synthesis of dibenzopyranones and pyrazolobenzopyranones through copper (0)/Selectfluor system-catalyzed double CH activation/oxygen insertion of 2-arylbenzaldehydes and 5-arylpyrazole-4-carbaldehydes:. Tetrahedron.

[ref17] (2020). coumarin-derived Fe 3+ Selective Fluorescence Sensor: Synthesis, Fluorescence Study and Application to Water Analysis: Scientific reports. Furo [3.

[ref18] (2019). A highly selective “Turn-on” fluorescent probe for detection of Fe 3+ in cells. Journal of fluorescence.

[ref19] (2019). Recent advances on iron (III) selective fluorescent probes with possible applications in bioimaging:. Molecules.

[ref20] (2016). Methylated urolithin A, the modified ellagitannin-derived metabolite, suppresses cell viability of DU145 human prostate cancer cells via targeting miR-21: Food and chemical toxicology.

